# Heterologous Expression of Laccase From *Lentinula edodes* in *Pichia pastoris* and Its Application in Degrading Rape Straw

**DOI:** 10.3389/fmicb.2020.01086

**Published:** 2020-05-26

**Authors:** Chanjuan Liu, Wenjing Zhang, Mingren Qu, Ke Pan, Xianghui Zhao

**Affiliations:** Jiangxi Province Key Laboratory of Animal Nutrition/Engineering Research Center of Feed Development, Jiangxi Agricultural University, Nanchang, China

**Keywords:** recombinant *Lentinula edodes* laccase, rape straw, lignin degradation, cellulase enzymolysis, soluble phenols

## Abstract

Rape straw cannot be efficiently degraded and utilized by ruminants due to its severe lignification and complex cross-linked structure between fiber and lignin. The laccases can catalyze the inter-unit bond cleavage in lignin substrates. Therefore, this study investigated the recombinant laccase from *Lentinula edodes* (LeLac) and its application in degrading rape straw. The LeLac was expressed using *Pichia pastoris.* It had the maximum activity at 60°C and pH 3.0 using ABTS as substrate and at 50°C and pH 4.0 using *o*-tolidine as substrate. The LeLac exhibited preferential oxidation of ABTS and featured resistance to high temperature, but relatively poor thermal stability. The LeLac activity could be strengthened by Cu^2+^ in dose-dependent manners. The LeLac could tolerate 15% of ethanol and methanol. The optimal pH for the lignin degradation of rape straw acid detergent fiber (ADF) by LeLac was 4.0. The LeLac could improve the cellulose enzymolysis of rape straw ADF by degrading its lignin. Relatively fewer lignin but more soluble phenols from original rape straw were removed by LeLac. The enhancement of enzymatic hydrolysis in original rape straw should be a combined result of polyphenols removal and lignin degradation caused by LeLac. This study demonstrated that the LeLac could improve the utilization of rape straw by degrading its lignin, meanwhile it’s worth noting that removing the soluble phenols by LeLac might also play an important role.

## Introduction

Though massive rape is grown in China every year, little of the rape straw produced is used efficiently and majority of it is burnt or naturally decayed as waste. Rape straw can theoretically be used as ruminants feed due to its high cellulose and hemicellulose contents, but severe lignification and complex cross-linked structure between fiber and lignin limit the penetration of ruminal microorganisms and their enzymes into plant cell wall (Ramirez-Bribiesca et al., 2011; Zhao et al., 2015). To improve the ruminal degradation of rape straw, it is necessary to disrupt the cross-linked structure and increase the surface area of available fiber. Treating with steam, alkali, acid, and so on has been shown to greatly destroy lignin and improve the digestibility of rape straw (Alexander et al., 1987). However, these methods show some unsatisfactory aspects, such as requiring expensive equipment, consuming great energy, endangering animal health, and damaging the environment, especially when alkali is used (Sarnklong et al., 2010; Li et al., 2019b).

It is generally known that the white-rot fungi could efficiently degrade lignocellulose in nature. The white-rot fungi *Lentinula edodes* could break down lignocellulose of rape straw and other agricultural straws by its enzymatic machineries and improve the ruminal utilization of cellulosic materials, but the hydrolysates are mainly used for its own growth, consequently causing the big losses of cellulose and hemicellulose (Mata and Savoie, 1998; Tuyen et al., 2012; Zhao et al., 2015). In addition, long incubation period was required for the degradation of rape straw by the natural development of *L. edodes*, which also limits its practical use. Our previous work observed the significant laccase activities during the cultivation of *L. edodes* using rape straw as substrate (Zhao et al., 2015). Laccases are multi-copper oxidases that catalyze the oxidation of a wide variety of aromatic substrates involving phenols, anilines and aromatic thiols, with the concomitant reduction of O_2_ to water (Alvira et al., 2013). Lignin is a polymer of aromatic subunits resulting from the oxidative combinatorial coupling of 4-hydroxyphenylpropanoids (Whetten and Sederoff, 1995; Vanholme et al., 2010). Studies suggested that laccases can catalyze polymerization of lignin or inter-unit bond cleavage in lignin substrates (Munk et al., 2015). Therefore, some researchers tried to remove or degrade lignin by laccase to improve the utilization of cellulose. Rai et al. (2019) reported that the doping of a highly thermostable recombinant laccase from *Geobacillus* sp. to commercial enzyme cocktails improved the hydrolysis of corn stover and bagasse. Pretreatment using laccase from *Sclerotium* sp. stimulated the cellulose conversion rate of steam blasting wheat straw no matter in the case of successive and simultaneous laccase and cellulase hydrolysis in the study by Qiu and Chen (2012). Rencoret et al. (2016) observed that *Pycnoporus cinnabarinus* laccase could significantly remove the lignin of wheat straw and consequently increase the glucose yields after enzymatic saccharification. Treating with laccase efficiently enhanced the digestibility of agricultural straws for ruminant feeding through delignification in the study by Kumar et al. (2018). Based on these reports, we hypothesize that the laccase from *L. edodes* (LeLac) could also enhance the degradation of rape straw lignin and consequent the enzymatic digestion of rape straw, however, little information is available. Therefore, this study expressed the LeLac using *Pichia pastoris* and evaluated its effects on the digestion of rape straw lignin and the enzymatic hydrolysis of rape straw treated by LeLac.

## Materials and Methods

### Synthesis of LeLac Gene and Construction of Expression Vector

The coding sequence of LeLac from *L. edodes*, GenBank accession AB035409.1, was optimized and synthesized by GenScript Nanjing, Co., Ltd. (Nanjing, China) according to Li et al. (2019a) (Supplementary Table S1). The synthetic DNA was ligated into pPICZαA, designating pPICZαA-LeLac. The pPICZαA-LeLac was transformed into *E. coli* DH5α by thermal shock, extracted, and verified according to our previous report (Li et al., 2019a).

### Transformation of *P. pastoris* and Screening of LeLac Expression Stain

The pPICZαA-LeLac was linearized using *Sac* I enzyme and transformed into competent *P. pastoris* X33 by electroporation (MicroPulser; Bio-Rad, Berkeley, CA, United States). The transformants were screened, certified, and grown in buffered glycerol-complex medium (BMGY) and methanol-complex medium (BMMY) successively according to Li et al. (2019a) to investigate their ability to secret LeLac. Yeast culture (1 ml) was collected every 24 h during methanol induction and centrifuged for the laccase activity analysis using 2,2′-azinobis-(3-ethylbenzothiazoline-6-sulfonic acid) (ABTS) as substrates (Zhao et al., 2015). The reaction mixture contained fermentation supernatants, 0.5 mM ABTS, and 0.1M citrate-Na_2_HPO_4_ buffer (pH 4.0). Supernatant with the highest activity was further confirmed by Western blot and the corresponding strain was selected and kept for the following laboratory scale incubation.

### Laboratory Scale Production of LeLac

The screened LeLac strain was grown in BMGY medium and then inoculated the staring volume (4.0 L of batch medium) of the bioreactor at 10% (v/v). The production was performed by a vertical glass bioreactor with 13.0-L volume (Labfors 5, Infors HT, Switzerland). The culture conditions were controlled at 30°C, pH 6.0, and more than 25% dissolved-oxygen concentration by cascadedly regulating the stirrer speed between 500 and 1,000 rpm and the airflow between 4 and 10 L h^–1^. The following incubation was carried out according to the *Pichia* fermentation process guidelines of Invitrogen. Sample was taken per day during methanol induction to analyze the activity. When the activity no longer increased, the incubation was stopped and supernatant was collected, centrifuged, and purified to obtain LeLac according to Li et al. (2019a). Briefly, the supernatant from centrifugation was concentrated using a membrane separation system (Jinan Bona Biological Technology, Co., Ltd., Jinan, China) and loaded on a 5 ml Bio-Scale Mini Nuvia IMAC Ni-Charged column (Bio-Rad, Berkeley, CA, United States) connected to a low-pressure chromatographic system (Biologic LP; Bio-Rad, Berkeley, CA, United States) to obtain the purified protein. The protein concentration was determined by a Bradford Protein Assay Kit (Sangon Biotech, Shanghai, China) and purity was confirmed by Sodium dodecyl sulfate polyacrylamide gel electrophoresis (SDS-PAGE) using a TGX Stain-Free FastCast Acrylamide Kit (Bio-Rad, Berkeley, CA, United States). The zymogram process was carried out using native-PAGE. Then the gel was incubated in 0.1M citrate-Na_2_HPO_4_ buffer, containing 0.5 mM ABTS (pH 3.0) and *o*-tolidine (pH 4.0) at room temperature until observing the enzyme band.

### Enzyme Activity Assays

The LeLac activity was determined by a reaction mixture containing 0.5 mM desired substrates and purified LeLac in 0.1M citrate-Na_2_HPO_4_ buffer according to the aforementioned description. The substrates involved ABTS, 2,6-dimethoxy phenol (DMP), *o*-tolidine, 3-aminobenzoic acid, *p*-phenylenediamine, veratryl alcohol, and guaiacol. One unit of enzyme activity (U) was defined as the amount of enzyme leading to the oxidation of 1 nmol of substrates per min per mg protein. The molar extinction coefficients (ε) of substrates and wavelength of determination were obtained from previous literature (Lee et al., 2012).

### Biochemical Characterization of LeLac

Determining the dependencies of pH for LeLac was carried out using purified enzyme, 0.5 mM ABTS/*o*-tolidine, and 0.1M glycine-HCL buffer (pH 2.0–3.0) or 0.1M citrate-Na_2_HPO_4_ buffer (pH 3.0–7.0) at 30°C. The effect of temperature on the LeLac activity was determined at 30–80°C with the optimum pH.

The thermal stability of LeLac was determined by incubating the enzyme in 0.1M citrate–phosphate buffer (optimum pH) for 0–24 h at 30, 40, 50, and 60°C and determining the remaining activity with time at optimum temperature using ABTS as the substrate.

The K_*m*_ and V_*max*_ for LeLac were determined in citrate-Na_2_HPO_4_ buffer (pH 3.0) containing 0–1.0 mM of ABTS at 60°C by a non-linear regression analysis using the Michaelis–Menten model with GraphPad Prism 8.0 (GraphPAD Software, Inc., San Diego, CA, United States). The turn over number (K_*cat*_) and catalytic efficiency (K_*cat*_/K_*m*_) of LeLac were determined using K_*m*_ and V_*max*_.

Five mM of MnCl_2_, CuSO_4_, KCl, NaCl, ZnCl_2_, MgCl_2_, CaCl_2_, and NiCl_2_ were used to determine the tolerance of LeLac to metal ions. Acetone, methanol, dimethyl sulfoxide (DMSO) and ethanol (15 and 30%) were used to analyze the tolerance of LeLac to organic solvents. The analyses were performed using ABTS as substrate under optimum conditions. The activity obtained without salts and organic solvents was defined as 100% and used as control. Other results were expressed as the percentage of control. To further investigate the effects of Cu concentration on the LeLac, 1–50.0 mM of CuSO_4_ were used according to the above description.

### Lignin Degradation and Cellulase Enzymolysis of Rape Straw Acid Detergent Fiber Treated by LeLac

The acid detergent fiber (ADF) of rape straw was prepared according to van Soest et al. (1991). It contained 68.9% cellulose, 29.9% lignin, and 1.2% ash. To determine the optimum pH for the lignin degradation of rape straw ADF, the study was carried out using 11.5 μg/mg substrate LeLac and 25 mg rape straw ADF in 2 ml 0.1M citrate-Na_2_HPO_4_ buffer (pH 3.0/pH 4.0) and shaking for 24 h at 30°C in triplicates. The control groups without LeLac were incubated similarly. To investigate the effects of LeLac concentration on lignin degradation, 0, 7.0, 11.5, and 16.0 μg/mg substrate purified LeLac were added into the reaction system (pH = 4) as described above.

To determine the synergism between LeLac and cellulase on the hydrolysis of cellulose in the rape straw ADF, the reaction was carried out using 4–48 μg/mg substrate cellulase (C1184, Sigma-Aldrich), 11.5 μg/mg substrate purified LeLac and 25 mg substrate in 2 ml 0.1M citrate-Na_2_HPO_4_ buffer (pH = 4) and shaken for 24 h at 30°C. The reducing sugars released in samples were determined using alkaline 3,5-dinitrosalicylic acid reagent.

### Lignin Degradation, Soluble Phenols Content, and Cellulase Enzymolysis of Original Rape Straw Treated by LeLac

The lignin degradation and cellulase enzymolysis of original rape straw treated by LeLac were analyzed according to above description by replacing rape straw ADF with rape straw. The mixtures contained 0 (control) or 11.5 μg/mg substrate purified LeLac and 48 μg/mg substrate cellulase. The total soluble phenols content was also determined when the study was finished.

### Analytical Procedures

The lignin content in samples was analyzed by a lignin assay kit (BC4200, Beijing Solarbio Science & Technology, Co., Ltd., Being, China). The ADF content of samples was determined according to van Soest et al. (1991). The samples were analyzed for ash by ashing at 550°C for at least 4 h. The cellulose content in samples was obtained by ADF content minus the content of lignin and ash. The total soluble phenols content was determined via the Folin-Ciocalteau method (Chen et al., 2015).

### Statistical Analyses

Statistical analysis was performed using a one-way ANOVA in IBM SPSS statistics version 20 (IBM, Chicago, IL, United States). Significance was declared at *P* ≤ 0.05.

## Results

### Production and Analysis of LeLac

**FIGURE 1:**
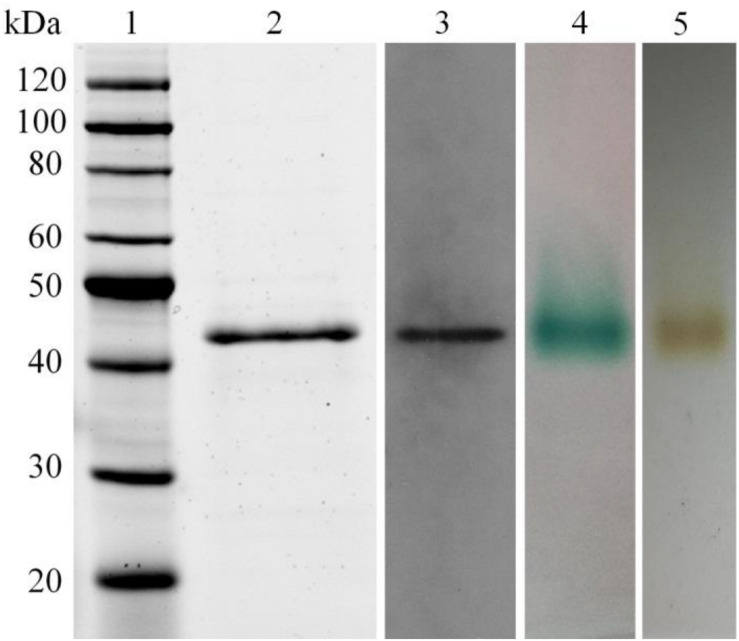
Analysis of recombinant LeLac by SDS-PAGE, Western blot, and zymogram. (1) Protein marker; (2) purified LeLac; (3) Western blot analysis of purified LeLac; (4) zymogram of purified LeLac using ABTS as substrate; (5) zymogram of purified LeLac using *o*-tolidine as substrate.

The LeLac gene was synthesized with codon optimization and expressed in *P. pastoris* to produce LeLac. One protein band at about 44 kDa was detected by SDS-PAGE, which was confirmed to be the interesting protein by Western blot analysis (Figure 1). The zymogram result revealed a single protein band corresponding to the position of LeLac activity (Figure 1).

### Enzyme Activity Assays of LeLac

**FIGURE 2:**
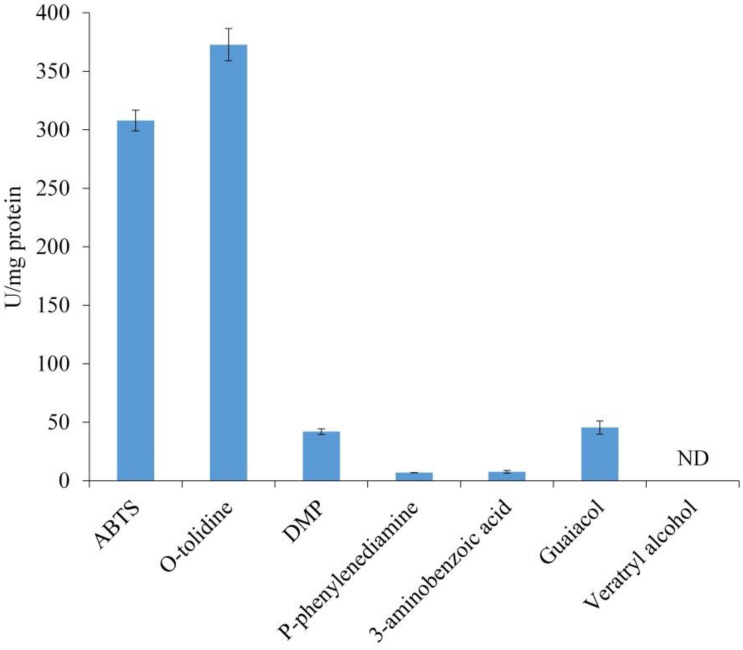
Enzyme activity assays of LeLac. Assays were carried out in 0.1M citrate-Na_2_HPO_4_ buffer (pH 4.0). One U was defined as the amount of enzyme leading to the oxidation of 1 nmol of substrates per min per mg protein. ND, none detectable.

**FIGURE 3:**
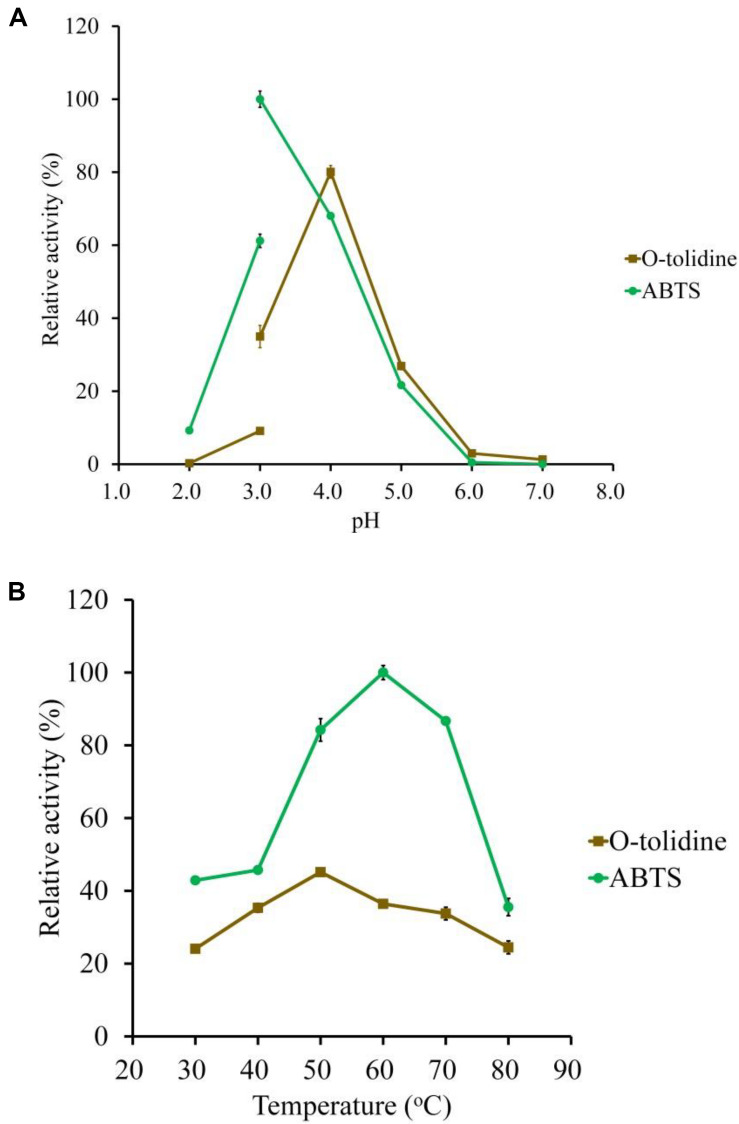
Determination of the optimal pH **(A)** and temperature **(B)** of the purified LeLac using ABTS and *o*-tolidine as substrates. The maximum activity obtained from ABTS was defined as 100%.

The substrate specificity of LeLac was examined by various phenolic and non-phenolic substrates. The LeLac exhibited maximum activity on *o*-tolidine, moderate activity on ABTS, slight activity on DMP and guaiacol, trifling activity on *p*-phenylenediamine and 3-aminobenzoic acid, and no significant activity was detected on 3, 4-dimethoxybenzyl alcohol (Figure 2). However, the results were related to the pH (pH 4.0) of buffer used. Latter data in this paper showed that the optimum pH was 4.0 and 3.0 for the oxidation of *o*-tolidine and ABTS by LeLac, respectively. The LeLac activity obtained using ABTS as substrate at pH 3.0 was higher than that obtained using *o*-tolidine as substrate at pH 4.0 (Figure 3A).

### Biochemical Characterization of Recombinant LeLac

ABTS and *o*-tolidine were used to determine the effects of pH and temperature on LeLac activity (Figure 3). The LeLac particularly favored acidic environment and exhibited maximal activity at pH 3.0 and pH 4.0 when the substrates were ABTS and *o*-tolidine, respectively (Figure 3A). The activity decreased sharply at pH 5.0–7.0 and achieve to an almost undetectable level as pH approached neutral regardless of substrate.

The LeLac showed maximum activity at 60 and 50°C as the substrates were ABTS and *o*-tolidine, respectively (Figure 3B). When the incubation temperature reached 70°C, the LeLac activity still retained 87 and 75% of maximal activity using ABTS and *o*-tolidine as the substrates, respectively.

**FIGURE 4:**
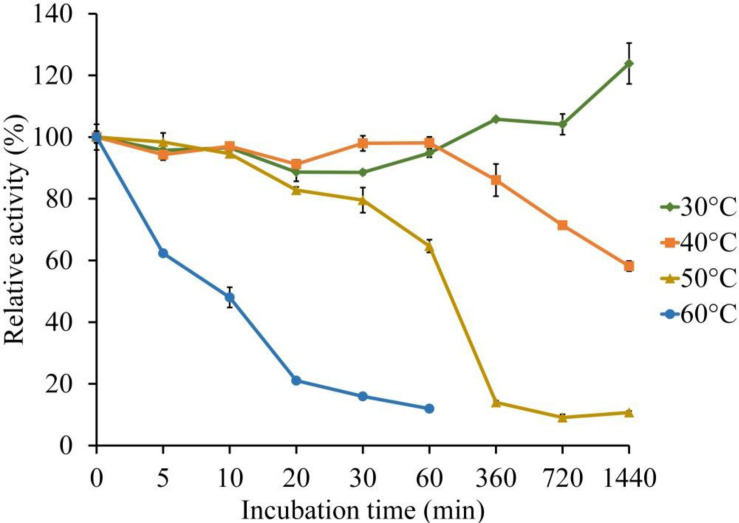
Thermal stability of LeLac determined using ABTS as substrate.

The thermal stability of LeLac was investigated by incubating the enzyme at 30, 40, 50, and 60°C for 0–24 h using ABTS as substrate in the present study. There was no activity loss after incubating at 30°C for 24 h, but incubating the enzyme at 40°C for 24 h, 50°C for 1 h, and 60°C for 10 min resulted in a 42, 35, and 52% loss of LeLac activity, respectively (Figure 4). When the LeLac was incubated at 60°C for 1 h, its residual activity was less than 15% of initial activity. Similar result was observed as the LeLac was incubated at 50°C for 6 h. Thus, we conclude that LeLac was very stable at 30°C and showed no sign of great thermal stability.

The results of kinetic parameters showed that the LeLac followed the Michaelis–Menten equation and exhibited K_*m*_ and V_*max*_ values of 16.82 μM and 13.97 μmol mg^–1^ s^–1^, respectively. According to the data, a K_*cat*_ value of 786.25 s^–1^ was calculated and the catalytic efficiency (K_*cat*_/K_*m*_) was found to be 46.74 μM^–1^ s^–1^.

**FIGURE 5:**
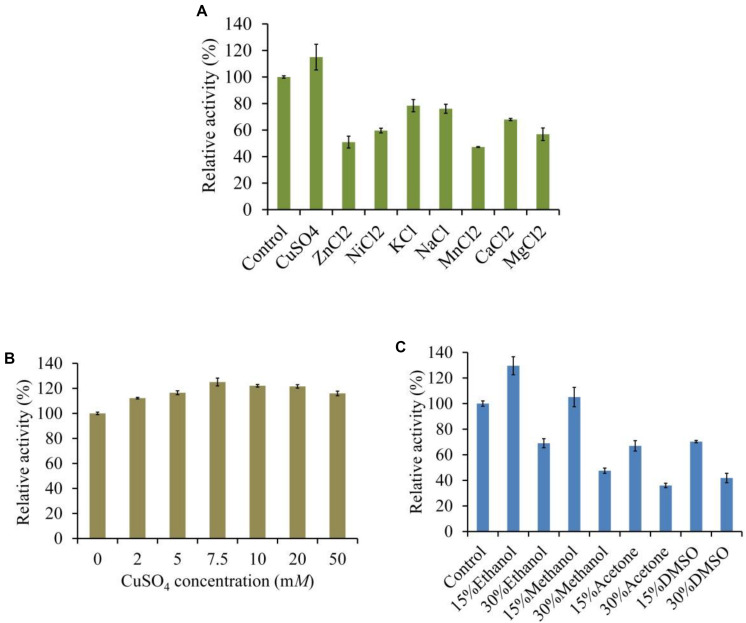
Effects of metal ions **(A)**, copper **(B)**, and organic solvents **(C)** on the activity of LeLac.

The LeLac activity was enhanced by 15% in the presence of 5 mM of Cu^2+^ but was reduced by other ions, especially Mn^2+^, which repressed 53% of activity (Figure 5A). We further investigated the effect of Cu^2+^ concentration on LeLac activity. The result suggested that Cu^2+^ affected LeLac activity by dose dependent manners and the maximum activity was observed as Cu^2+^ concentration was 7.5 mM (Figure 5B). Though 10–50 mM of Cu^2+^ repressed the LeLac activity compared with 7.5 mM of Cu^2+^, the enzyme activity was still greater than that without Cu^2+^ addition.

The LeLac activity was increased by 30 and 5% in the presence of 15% (v/v) of ethanol and methanol, respectively (Figure 5C). When the solvent concentration arrived at 30%, all the organic solvents used inhibited the LeLac activity.

### Lignin Degradation and Cellulase Enzymolysis of Rape Straw ADF Treated by LeLac

**FIGURE 6:**
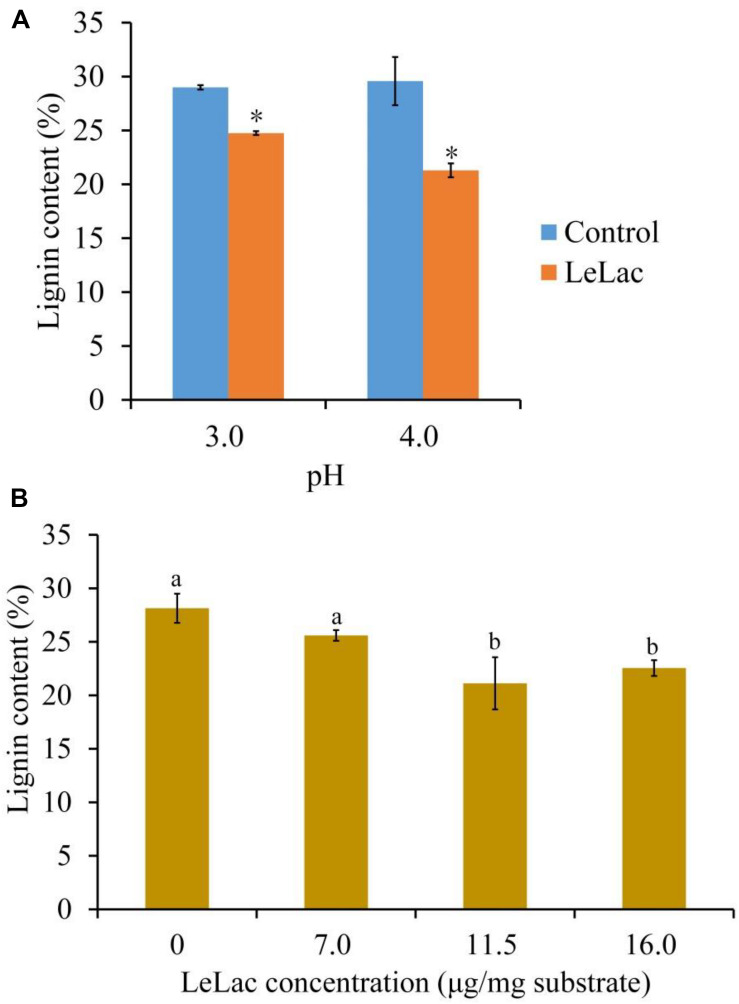
Effects of reaction pH **(A)** and dosage **(B)** on the lignin degradation of rape straw ADF caused by LeLac. Different letters indicate significant difference for the same enzyme concentration. Asterisks indicate significant differences (*P* ≤ 0.05) for the same pH. Different letters indicate significant difference (*P* ≤ 0.05).

Given that the optimal pH for LeLac activity was substrate-dependent, the current study investigated the optimal pH for the lignin degradation of rape straw ADF by LeLac. It was shown that the lignin content of rape straw ADF were 24.8 and 21.3% after LeLac treating at pH 3.0 and 4.0, respectively, indicating pH 4.0 was optimal for the lignin degradation of rape straw ADF by LeLac (Figure 6A).

**FIGURE 7:**
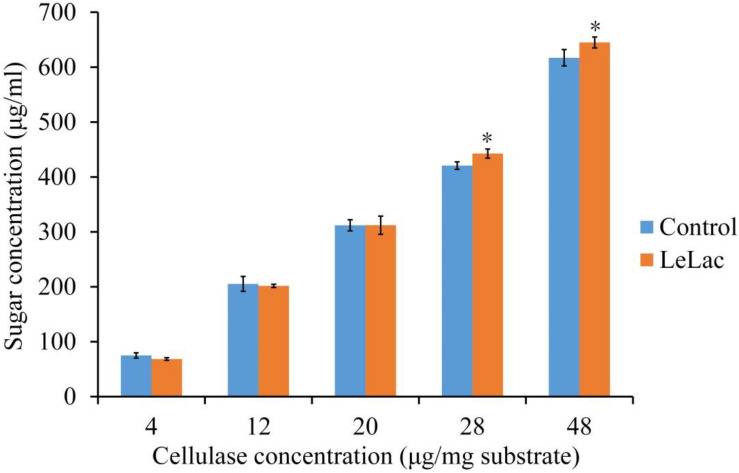
The synergism between LeLac and cellulase on the hydrolysis of cellulose in the rape straw ADF. Asterisks indicate significant differences (*P* ≤ 0.05) for the same cellulase concentration.

The effects of LeLac concentration on lignin degradation of rape straw ADF were detected in the present study. The results showed that the lignin contents were reduced by 2.5, 7.0, and 5.6% for the addition of 7.0, 11.5, and 16.0 μg/mg substrate of LeLac (Figure 6B). Then this study further investigated the effect of LeLac treating on cellulose enzymolysis of rape straw ADF. The results showed that the reducing sugars contents of LeLac groups were increased by 21.9 and 27.8 μg/ml relative to the control groups when the cellulase concentrations were 28.0 and 48.0 μg/mg substrate, respectively, indicating that LeLac treatment enhanced the cellulose hydrolysis of rape straw ADF (Figure 7). When the cellulase concentration was low (4.0–20.0 μg/mg substrate), however, the reducing sugars yield was not different between the control and LeLac groups. This may be because that the most digestible fraction of rape straw ADF was enough for the binding and degradation of low doses of cellulase, as a result, the advantage resulting from lignin removal was not shown during cellulase enzymolysis.

### Lignin Degradation, Soluble Phenols Content, and Cellulase Enzymolysis of Original Rape Straw Treated by LeLac

**FIGURE 8:**
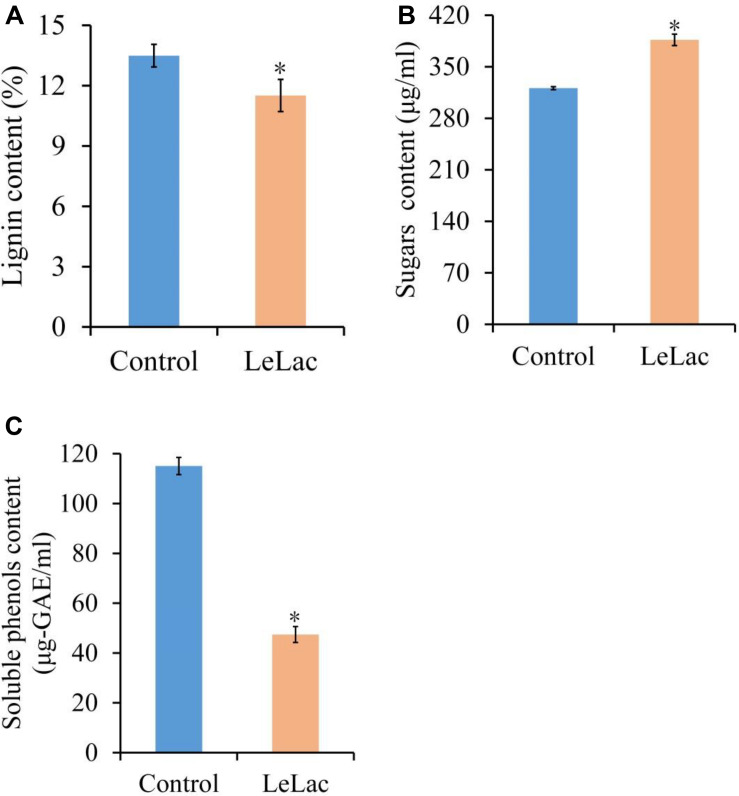
Effects of LeLac on the lignin content **(A)**, cellulase hydrolysis **(B)**, and soluble phenols content **(C)** of the original rape straw. The mixtures contained 0 (control) or 11.5 μg/mg substrate of purified LeLac and 48.0 μg/mg substrate of cellulase. Asterisks indicate significant differences (*P* ≤ 0.05).

The lignin content of original rape straw was reduced by 2.0% after LeLac treating (Figure 8A). The degree of decrease was lower than that using rape straw ADF (7.8%), though the reaction environment was the same. However, the reducing sugars content was increased by 65.6 μg/ml for the LeLac-treated group compared with the control group (Figure 8B), which was greater than that obtained from rape straw ADF (27.8 μg/ml). No soluble phenols were detected for rape straw ADF (data not shown), however, there was 115.1 μg-gallic acid equivalents (GAE)/ml soluble phenols for original rape straw after 24-h incubation (Figure 8C). The soluble phenols content was reduced to 47.4 μg-GAE/ml after LeLac treating.

## Discussion

To improve the utilization of rape straw, a recombinant laccase from *L. edodes* was produced using *P. pastoris*, and its characteristics and effects on rape straw hydrolysis were investigated in current study. The results showed that the optimal pH for the LeLac activity was substrate-dependent and was pH 3.0 and pH 4.0 when the substrates were ABTS and *o*-tolidine, respectively, which may be attributed to variable degrees of substrate protonation under different pH conditions (Nagai et al., 2002). The LeLac activity was conspicuously suppressed under pH 5.0 and was almost inactive when the pH exceeded 6.0, which may be due to the redox potential difference between the reducing substrate and the type 1 copper in the active site of laccase and the competitive binding of hydroxide anion to the type 2/type 3 coppers of laccase at higher pH (Xu, 1997). The optimal pH range for laccases from other fungal was from 3.0 to 6.0 in many studies (Sadhasivam et al., 2008; Lee et al., 2012). Similar to the present study, the optimum pH for a laccase isolated from *L. edodes* was around pH 4.0 in the study by Nagai et al. (2002).

The LeLac activity was still over 75% of maximal activity at 70°C in the present study, suggesting that the LeLac could resist high temperature. However, the LeLac did not have excellent thermal stability and could be inactivated at 60°C for more than 1 h. The optimal reaction temperature of laccases varies considerably depending on the source organism. The laccase from *Trichoderma harzianum*, *Rigidoporus lignosus*, and *Yarrowia lipolytica* had the maximum activity at 35, 40, and 70°C, respectively (Cambria et al., 2000; Sadhasivam et al., 2008; Lee et al., 2012). Similar to the present recombinant laccase, the laccase isolated and purified during *L. edodes* cultivation also lost more than 90% of activity after incubating at 60°C for 30 min (Nagai et al., 2002).

The metal ions could affect the LeLac activity in the present study, which was obviously enhanced in the presence of Cu^2+^. Copper could induce the protein expression and activity enhancement of laccase during many white rot fungi cultivation (Palmieri et al., 2000; Galhaup and Haltrich, 2001; Baldrian and Gabriel, 2002; Fonseca et al., 2010). The experiment by Baldrian and Gabriel (2002) with purified laccase from *Pleurotus ostreatus* showed that Cu^2+^ (0.05–50.0 mM) not only induced the expression of laccase genes, but it also enhanced the activity and stability of the enzyme. Lu et al. (2009) also observed that 0.1–0.5 mM of Cu^2+^ significantly increased the activity of recombinant laccase from *P. pastoris*. In addition, Cu^2+^ also enhanced the activity of some bacterial laccases in the previous studies (Berini et al., 2018; Rai et al., 2019). The current results coincided with those reports. Laccase is copper-containing enzyme and its activity is directly proportional to the copper concentration in the growth medium or reaction system, indicating the importance of copper for the enzyme (Claus, 2004). The LeLac activity was inhibited in the presence of Zn^2+^, Ni^2+^, K^+^, Na^+^, Mn^2+^, Ca^2+^, and Mg^2+^ in the current study. Rai et al. (2019) found that the activity of a recombinant laccase from *Geobacillus* sp. was repressed by 1 mM of Zn^2+^, K^+^, Na^+^, Mn^2+^, Ca^2+^, and Mg^2+^. Murugesan et al. (2009) reported that the purified laccase of *Ganoderma lucidum* was sensitive to 5 mM of Ni^2+^, K^+^, Na^+^, Mn^2+^ but was activated by Zn^2+^ and Ca^2+^. The activity of purified laccase of *Streptomyces psammoticus* was inhibited by 2 mM of Mn^2+^ and Ca^2+^, but was enhanced by K^+^, Na^+^, Mg^2+^, and Zn^2+^ in the study by Niladevi et al. (2008). Hu et al. (2014) observed that the activity of laccase from *Leptographium qinlingensis* was restrained by 1 and 10 mM of K^+^, Mn^2+^, Ca^2+^, and Mg^2+^. Current study was partial agreement with the above reports. Those results were also indicated that the tolerant activity of laccase toward metal ions depended on the source of organism. Two proposed theories have been used to explain the effects of metal ions on laccase activity. When metal ions enhanced the laccase activity, the binding of these metal ions induces conformational modification of the enzyme and stimulates decomposition of the complex containing substrate, enzyme and metal ions, but when metal ions inhibited the laccase activity, these metal ions maybe binds near the type I copper site of laccase and acts as a competitive inhibitor of electron donors by inhibiting the access of substrates to the site or the electron transfer at the site (Si et al., 2013).

Many substrates for enzymes as well as enzymolysis products dissolve only in organic solvents and their analytical assay requires the application of non-aqueous media, which increased the interest in studying function and activity of enzyme in organic solvents (Bogdanovskaya et al., 2002). The current results showed that the LeLac could tolerate low-dose (15%) ethanol and methanol. Similarly, the activity of laccase from *Kurthia huakuii* was enhanced in the presence of 10% of ethanol and methanol in the study by Guo et al. (2016). Mozhaev et al. (1989) reported that there was a critical concentration of ethanol (approximately 37%) affecting laccase activity, after which the laccase activity dramatically decreases due to its denaturation. Bogdanovskaya et al. (2002) pointed out that the inhibiting effect of solvents on laccase maybe resulted from the competing binding of organic solvent to the active center of laccase. In addition, the organic solvents used in the current study were all polar, which maybe took crucial bound-water from the enzyme molecule essential for its activity (Ogino and Ishikawa, 2001). Similarly, some laccases from previous studies were also instable in water-polar solvents mixtures (Rodakiewicz-Nowak et al., 1999, 2000).

The organic compounds contained in the rape straw ADF were only cellulose and lignin. Therefore, to investigate the effect of LeLac on lignin degradation and cellulose hydrolysis after lignin removal of rape straw, rape straw ADF, the relatively simple substance relative to the original rape straw, was used in the current study. As expected, the LeLac treating resulted in the reduction of lignin content of rape straw ADF. Laccase was an important part of ligninolytic enzyme system (i.e., laccase, manganese peroxidase, lignin peroxidase) from white rot fungi (Youn et al., 1995; Eggert et al., 1997; Manavalan et al., 2015). Unlike manganese peroxidase and lignin peroxidase which require Mn^2+^ and H_2_O_2_ as the electron donor, respectively, laccase could directly catalyze the substrate oxidation with a concomitant four-electron reduction of molecular oxygen to water (Manavalan et al., 2015), making it have a better application potential. Many studies observed the delignification activity of laccase from different microorganism on agricultural straws (Qiu and Chen, 2012; Rencoret et al., 2016, 2017; Kumar et al., 2018). The pretreatment using laccase with/without mediator removed up to 1.6–48% of lignin from straws in these studies, suggesting that the LeLac could efficiently degrade the lignin of rape straw like the aforementioned laccases. After the removal of lignin by LeLac, the cellulase hydrolysis of cellulose in rape straw ADF was enhanced in the current study. The cellulose and hemicellulose of plants are trapped within the lignin matrix resulting in the poor hydrolysis by cellulolytic enzyme. Application of lignin degrading enzymes on the plant biomass could expose the cellulose for the access of cellulolytic enzyme. Therefore, many studies tried to improve the hydrolysis or saccharification by removing lignin using laccase and obtained positive results (Sitarz et al., 2013; Moreno et al., 2015). For example, the glucose yield of wheat straw was increased from 24.1 to 33.6% with the reduction of lignin content from 15.6 to 9.8% caused by *Pycnoporus cinnabarinus* laccase-mediator treatment in the study by Rencoret et al. (2016). Another report of these authors found that the laccase-mediator pretreatment decreased the lignin content by 4.7 and 5.1% and correspondingly increased the glucose release by around 15.7 and 17.9% for sugarcane bagasse and straw, respectively (Rencoret et al., 2017). Some *in vitro* studies showed that supplemental laccase improved the ruminal digestibility of straw fiber up to 25% compared with the control group (Si et al., 2015; Kumar et al., 2018). Current results supported the previous reports. This positive effect of the laccase on cellulase hydrolysis is attributed to the increase of cellulose exposed and the reduction of the non-specific adsorption of cellulase to lignin by delignification or/and increasing the amounts of carboxylic acids, which specifically reduced the non-specific adsorption of negatively charged cellulase (Palonen and Viikari, 2004; Rencoret et al., 2017).

The physical structure and chemical composition of original rape straw were far more complex than those in rape straw ADF. Therefore, this study further investigated the effects of LeLac on original rape straw. The results showed that though the LeLac removed less lignin from original rape straw, it obviously enhanced the cellulase hydrolysis of cellulose compared with the rape straw ADF. It’s understandable that more complex structure and chemical composition in the original rape straw possibly repressed the degradation of LeLac on lignin, however, the disproportion between lignin removal and increased cellulase hydrolysis for original rape straw was interesting. Therefore, the present study investigated the soluble phenols content of original rape straw with/without LeLac treating. Many studies reported the strong inhibition of phenolic molecules on the activity of cellulases (Kim et al., 2011; Tejirian and Xu, 2011; Ximenes et al., 2011; Qin et al., 2016). These phenolics could inactivate cellulases by complexing them and inhibit enzymatic cellulolysis by adsorbing onto cellulose (Tejirian and Xu, 2011). As a soluble polyphenol, tannins could interact with proteins and decrease glucosidase activity from almonds due to irreversible protein denaturation (Kawamoto et al., 1997). Previous studies showed that laccase could efficiently remove polyphenols and consequently enhanced the enzymatic hydrolysis of substrates and reducing sugars yield (Lee et al., 2012; Kalyani et al., 2015). After LeLac treating, the soluble phenols and reducing sugars were reduced by 58.8% and increased by 20.5%, respectively, in the present study, which coincided with the previous reports. And then, current study indicated that the enhancement of enzymatic hydrolysis in original rape straw should be a combined result of polyphenols removal and lignin degradation caused by LeLac based on the preceding sections.

## Conclusion

The LeLac was expressed using *P. pastoris* and shown maximum activity at 60°C and pH 3.0 using ABTS as substrate and at 50°C and pH 4.0 using *o*-tolidine as substrate. LeLac did not possess good thermal stability. The optimal pH for LeLac was 4.0 when the substrate was rape straw ADF. The LeLac activity was strengthened by Cu^2+^ in dose dependent manners. The LeLac could improve the cellulose enzymolysis of rape straw ADF by degrading its lignin. The soluble phenols from original rape straw were efficiently removed by LeLac. The enhancement of enzymatic hydrolysis in original rape straw should be a combined result of polyphenols removal and lignin degradation caused by LeLac. The LeLac should be useful for improving the utilization of rape straw, and it’s worth noting that removing the soluble phenols by LeLac might play an important role.

## Data Availability Statement

All datasets generated for this study are included in the article/Supplementary Material.

## Author Contributions

XZ designed the study, wrote the manuscript, and primary responsibility for the final content. CL and WZ conducted the study and collected the data. CL, WZ, and KP analyzed the data. MQ helped with the manuscript writing. All authors have read and approved the final manuscript.

## Conflict of Interest

The authors declare that the research was conducted in the absence of any commercial or financial relationships that could be construed as a potential conflict of interest.

## Supplementary Material

TABLE S1 Optimized and original coding sequence of *L. edodes* laccase (GenBank accession No. AB035409.1).
