# Supplementary material for: Heterologous Expression of Laccase From Lentinula edodes in Pichia pastoris and Its Application in Degrading Rape Straw
Source: Front Microbiol. 2020 May 26;11:1086. doi: 10.3389/fmicb.2020.01086 (PMC7264821; doi:10.3389/fmicb.2020.01086)
Supplement: TABLE S1 — Optimized and original coding sequence of L. edodes laccase (GenBank accession No. AB035409.1). [file Table_1.DOCX]

***Lentinula edodes* laccase sequence**

**Original Sequence**

GCCATCGGTCCTGTCACTGACTTGCATATCGTGAACTCGTTCATTCAACCTGACGGCTTCAACCGCTCTGGTGTCCTTGCTGAGGGCGTCTTTCCTGGACCACTCATCACGGGTAACAAAGGCGACAATTTCCAGATCAATGTGATTGATGAGCTCACTAACGGAACGATGCTCTTGAGCACTTCGATTCATTGGCACGGCCTTTTTCAGAAAACAACCAACTGGGCAGACGGACCAGCATTCGTGAATCAATGTCCTATCGCCGCGAATGATTCCTTCCTGTACAATTTCAATGTTCCGGACCAAGCTGGGACTTTCTGGTATCACAGTCACCTATCCACTCAATATTGCGATGGATTGCGTGGACCCTTGGTGGTGTATGACCCTCAAGATCCATACGCTGACCTATACGACGTTGATGATGATTCAACTGTCATCACACTCGCTGACTGGTATCACGTTCCCGCCCCTCAAGCTGGCGCTGTTCCGACCTCGGACGCCACTCTCATCAATGGCCTAGGACGATCCGTCAACGGTCCAGCCGATGCACCCTTTGCTGTAGTCAATGTCGTGCAGGGTAGTCGCTATCGTTTTCGTTTGGTGTCGATATCTTGTGATCCGAACTTCTTGTTTTCGATTGACGGGCACACCTTTACTGTCATTGAAGCTGATGGTGTTAATCATGAACCCATTGTCGCTGATTCCATCCAGATTTTCGCTGCTCAACGATACTCGTTCATTCTCACTGCAAATCAAACTGCTGATAATTATTGGATCCGTGCCAATCCTAACAACGGACATACCGGCTTCGCTGGGGGTATAAACAGCGCGATTCTGCGTTATTCTGGCGCCCCTGTTGCTGATCCGGTCACTACCCAGACTTCTGCCAACCTTCTACAGGAAACAAGCCTTGTGCCGCGTGAAAATCCCGGTGCTCCTGGAAATGCAACTGCTAACGGCGTAGATGTTGACTTGAATCTGGTCTTATCATTTGTTGGAGGACGGTTCGAGATTAACGGGGTATCCTTTGTACCCCCTACAGTCCCTGTCTTACTTCAAATTCTCAGCGGAGCCACTACAGCAGCCGAACTTCTACCCAGTGGCTCTGTTTATACATTGCCCTTGAATTCGGTCATCCAGTTGAGCTTTAATACGGTTGCAGTTGCAGCAGTTGGAGGTCCTCATCCATTCCATCTTCACGGGCACACATTTGACGTGGTCCGAAGTGCCGGAAGTACCGAGTATAACTATATCAACCCTCCGCGCAGGGATGTCGTCTCTACTGGTGCAGCTACTGACAATGTAACCATTCGTTTTACTACTGATAATGCTGGACCATGGTTCCTTCACTGTCACATAGACTGGCATCTGGAAGCTGGTTTTGCTATTGTTTTTGCTGAGGATGCACCGGACGTCGCCGCCGTTAATCCCGTCCCCGACGCTTGGAATCAACTTTGCCCAACTTATGATGCTTTGACCCCTGCACAACTTGGTGGAAAT

**Optimized Sequence**

GAATTCGCTATTGGTCCAGTTACTGATTTGCATATCGTTAATTCTTTTATTCAACCTGATGGTTTCAACAGATCTGGTGTTTTGGCTGAAGGTGTTTTTCCAGGTCCTTTGATCACTGGTAACAAGGGAGATAACTTCCAAATTAACGTTATCGATGAGTTGACTAACGGTACTATGTTGTTGTCTACTTCTATTCATTGGCACGGTTTGTTTCAAAAGACTACTAATTGGGCTGATGGTCCAGCTTTCGTTAATCAATGTCCTATTGCTGCTAACGATTCTTTCTTGTACAACTTCAATGTTCCAGATCAAGCTGGTACTTTTTGGTACCATTCTCACTTGTCTACTCAATATTGTGATGGTTTGAGAGGTCCTTTGGTTGTTTACGATCCACAAGATCCTTACGCTGATTTGTACGATGTTGATGATGATTCTACTGTTATTACTTTGGCTGATTGGTATCACGTTCCAGCTCCTCAAGCTGGTGCTGTTCCAACTTCTGATGCTACTTTGATTAATGGTTTGGGTAGATCTGTTAACGGTCCAGCTGATGCTCCTTTTGCTGTTGTTAATGTTGTTCAAGGTTCCAGATACAGATTCAGATTGGTTTCTATCTCTTGTGATCCAAACTTTTTGTTCTCTATTGATGGTCACACTTTTACTGTTATTGAAGCTGATGGTGTTAATCATGAGCCTATCGTTGCTGATTCTATCCAAATCTTCGCTGCTCAAAGATACTCTTTCATTTTGACTGCTAATCAAACTGCTGATAACTATTGGATTAGAGCTAATCCAAACAATGGTCATACTGGTTTCGCTGGTGGTATTAACTCTGCTATTTTGAGATATTCTGGTGCTCCAGTTGCTGATCCTGTTACTACTCAAACTTCTGCTAATTTGTTGCAAGAAACTTCTTTGGTTCCTAGAGAGAACCCAGGTGCTCCTGGTAATGCTACTGCTAACGGTGTTGATGTTGATTTGAATTTGGTTTTGTCTTTTGTTGGTGGTAGATTCGAAATTAACGGTGTTTCTTTTGTTCCACCTACTGTTCCAGTTTTGTTGCAAATTTTGTCTGGTGCTACTACTGCTGCTGAGTTGTTGCCATCTGGTTCTGTTTACACTTTGCCTTTGAACTCTGTTATTCAATTGTCTTTCAACACTGTTGCTGTTGCTGCTGTTGGTGGTCCACACCCTTTTCATTTGCACGGTCATACTTTCGATGTTGTTAGATCTGCTGGTTCTACTGAATACAACTATATTAATCCACCTAGAAGAGATGTTGTTTCTACTGGTGCTGCTACTGATAACGTTACTATCAGATTCACTACTGATAACGCTGGTCCATGGTTCTTGCACTGTCATATTGATTGGCATTTGGAAGCTGGTTTTGCTATTGTTTTCGCTGAGGATGCTCCTGATGTTGCTGCTGTTAATCCAGTTCCTGATGCTTGGAACCAATTGTGTCCAACTTATGATGCTTTGACTCCTGCTCAATTGGGTGGTAACTTTCTAGA
